# Supplementation of Mother’s Own Milk with Donor Milk in Infants with Gastroschisis or Intestinal Atresia: A Retrospective Study

**DOI:** 10.3390/nu12020589

**Published:** 2020-02-24

**Authors:** Rebecca Hoban, Supriya Khatri, Aloka Patel, Sharon L. Unger

**Affiliations:** 1 Department of Paediatrics, Division of Neonatology, Hospital for Sick Children, Toronto, ON M5G 1X8, Canada; supriya.khatri@sickkids.ca (S.K.); sharon.unger@sinaihealthsystem.ca (S.L.U.); 2 Faculty of Medicine, University of Toronto, Toronto, ON M5S 1A8, Canada; 3 Department of Pediatrics, Division of Neonatology, Rush University Children’s Hospital, Chicago, IL 60612, USA; Aloka_Patel@Rush.edu; 4 Department of Paediatrics, Sinai Health, Toronto, ON M5G 1X5, Canada

**Keywords:** human milk, donor milk, neonatal, gastroschisis, intestinal atresia

## Abstract

Background: Mother’s own milk (MOM) improves in-hospital outcomes for preterm infants. If unavailable, donor milk (DM) is often substituted. It is unclear if DM vs. formula to supplement MOM is associated with improved in-hospital outcomes in term/late preterm surgical infants with gastroschisis or intestinal atresia. Methods: This retrospective study included infants born ≥33 weeks gestational age (GA) with a birth weight of >1500 g who were admitted to a quaternary neonatal intensive care unit (NICU). Using Chi square and Mann-Whitney u testing, we compared hospital outcomes (length of stay, parenteral nutrition and central line days) before and after a clinical practice change to offer DM instead of formula in this surgical population. Results: Baseline characteristics were similar between eras for the 140 infants (median GA 37 weeks). Fewer infants in DM era were receiving formula at discharge (50.0% vs. 31.4%, *p* = 0.03). In sub-analyses including only small bowel atresia and gastroschisis infants, the median length of stay (35 vs. 25, *p* < 0.01) and the central line days (28 vs. 20, *p* < 0.01) were lower in the DM era. Conclusion: In this retrospective study, offering DM instead of formula was associated with less formula feeding at discharge, and in infants with gastroschisis or small bowel atresia, shorter length of stay and central line days.

## 1. Introduction

Infants born with congenital malformations of the gastrointestinal (GI) tract, specifically those with gastroschisis or intestinal atresia, require surgical intervention early in life. Their abnormal GI tract necessitates prolonged parenteral nutritional (PN) [1,2] support prior to and after surgery, which puts these infants at risk of morbidities such as central line-associated sepsis and PN-induced cholestasis. In addition, once enteral feedings are initiated these infants commonly experience feeding intolerance and secondary morbidities such as necrotizing enterocolitis (NEC) [1,3] because of bowel exposure to inflammatory amniotic fluid and/or distension during fetal life [3]. All of these challenges, combined with the fact that these infants are often born late preterm [2], result in prolonged courses of PN and long hospital length of stays (LOS). Human milk (HM), which is most often mother’s own milk (MOM), reduces the incidence of NEC in high-risk populations such as infants with preterm birth or gastroschisis [4,5,6]. Additionally, HM has been reported to shorten LOS, PN time and time to full enteral feeds in congenital GI malformations requiring surgery (gastroschisis, atresia) [7,8]. HM also reduces PN time and PN-associated liver disease in infants with short bowel syndrome [9,10] or intestinal failure, which may be a consequence of congenital GI malformations. Infants with congenital GI malformations are nothing by mouth (NPO, nil per os), are separated from their mothers after birth, and are often late preterm, requiring their mothers to initiate lactation exclusively with a breast pump, resulting in lactation challenges, risks of poor MOM supply [11,12,13,14,15] and lower rates of breastfeeding. If a supplement to MOM is required, the current standard of care in the term/late preterm population is the use of formula.

In other high-risk populations, namely very preterm (<32 weeks gestation) infants, pasteurized donor human milk (DM) is now commonly used if exclusive MOM is unavailable. Pasteurization and multiple freeze thaw cycles result in a product quite different than MOM in a myriad of ways, from bioactive to nutritional properties [16], but in the preterm population DM use avoids the risks of formula, specifically NEC [17]. Since DM is an expensive and limited resource, most Neonatal Intensive Care Units (NICUs) limit DM provision to very preterm infants, excluding most infants with congenital GI malformations. There are no trials of its efficacy in infants with congenital GI malformations, although it has been suggested that DM could have benefit in this population based on extrapolation from the preterm infant literature [12].

In 2013, the Hospital for Sick Children (HSC), in conjunction with the Rogers Hixon Ontario Human Milk Bank, changed its practice from only supplying DM to very preterm infants to also offer DM in-hospital to infants with congenital GI malformations in the first month post-surgery if MOM supply was inadequate. Given the limited literature, we sought to determine whether the use of DM for infants with congenital GI malformations who would not otherwise qualify for DM was associated with improved outcomes after this practice change. Primary outcomes included days of PN and central venous line (CL) as well as LOS. Secondary outcomes included comparing the use of MOM in both eras as a balancing measure, as offering DM could affect MOM provision [16]; comparing growth in both eras, as DM in the preterm population has been variably associated with poor growth [16,18]; and to compare risks of NEC (medical or surgical), culture positive sepsis and death in the pre-DM and DM eras.

## 2. Materials/Subjects and Methods

This was a retrospective single center study that included neonates admitted to an urban, quaternary outborn neonatal intensive care unit (NICU) in Toronto, Canada (the Hospital for Sick Children) prior to any surgical intervention who held a diagnosis of gastroschisis or congenital intestinal atresia.

### 2.1. Sample

Eligible infants had a birthweight (BW) >1500 g and/or gestational age (GA) at birth ≥33 weeks with a diagnosis of gastroschisis and/or congenital lower intestinal atresia (duodenal, jejunal, or ileal atresia or colonic/rectal atresia without a fistula). Included infants in this convenience sample were born between 1 July 2011 and 31 December 2012 (pre-DM era) and between 1 January 2014 and 31 May 2015 (DM era) to encompass approximately equal eras surrounding a practice change to use DM in late preterm or term surgical infants in early 2013. 2013 was excluded as a “wash-out” year to ensure complete separation between groups given long LOS and to allow for clinical uptake of the practice change. Of note, feeding initiation and advancement guidelines were slightly modified at our institution in early 2011 (prior to the study) and did not change for the duration of the study. Term and late preterm infants with congenital GI malformations would typically receive unfortified MOM or term formula; DM had protein powder added to approximate typical MOM protein levels. Infants would only receive bovine-based HM fortifiers for clinical concerns of poor growth once full enteral feeding tolerance was achieved. Infants were excluded if their initial admission to the NICU occurred after surgical intervention (i.e., transferred from another tertiary institution for second opinion or continued care). Other congenital gastrointestinal surgical conditions such as omphalocele, esophageal atresia, and anorectal malformation with fistula were not included unless co-existing with an included condition (such as both duodenal atresia and omphalocele). Lactation consultation and support was available to all mothers, as was a hospital grade double electric pumps for use in-hospital or to borrow without cost for home use. DM was offered after the practice change with parental consent when MOM supply was inadequate for the first post-operative month or until nearing discharge, whichever occurred sooner, with gradual weaning to formula occurring over a 48 h period.

### 2.2. Design and Measures

This study was approved by the institutional Research Ethics Board at the Hospital for Sick Children, and given its retrospective nature, was exempted from consent. Data were extracted from the electronic medical records for both eras after relevant medical record numbers were pulled from an internal NICU electronic database based on the study inclusion criteria of diagnosis, birthweight, gestational age and date of birth. Relevant electronic medical records were then hand searched to obtain and confirm each subject’s relevant surgical primary (and secondary, if applicable) diagnosis, birth GA (completed weeks), sex, and birth anthropometrics (BW in grams, length and head circumference in cm). Outcomes collected included corrected GA at discharge, discharge anthropometrics (weight, length and head circumference), total days on PN, total days with a CL (peripherally inserted and/or surgically placed central catheter and/or umbilical venous catheter) during hospital stay (intermittent days summed if not continuous), hospital length of stay (LOS; days) and the dichotomous outcomes of any use of MOM, any use of DM and any use of formula during hospital stay and when this occurred (≤30 days of life vs. >30 days of life), MOM use in the 48 h prior to hospital discharge, death before discharge, NEC (if yes, medical or surgical) and culture positive blood steam infection. Data were de-identified upon chart extraction.

### 2.3. Data Analysis

Study data were collected and managed using REDCap electronic data capture tools [19] hosted at the Hospital for Sick Children and were analyzed using SPSS version 26 (IBM, Armonk, NY, USA). Descriptive statistics were used to summarize sample characteristics, and after testing for normalcy, pre-DM and DM infants were compared using t-testing, Mann-Whitney u testing or chi-square. Large diagnostic groups (i.e., gastroschisis and atresia) were studied as a whole as well as separately to assess for different signals, specifically focusing on small bowel atresias, which commonly exhibit prenatal bowel distension unlike large bowel atresias [2,15] and gastroschisis, in which the bowel may also exhibit distension in addition to being subjected to irritating amniotic fluid in the latter case. If an infant had both gastroschisis and intestinal atresia, gastroschisis was considered the primary diagnosis and dictated diagnostic grouping.

## 3. Results

### Characteristics of the Sample

Infants (*n* = 167) were initially identified in an electronic search using the inclusion criteria. After manually extracting all data from the electronic record, 27 were excluded due to a diagnosis of colonic/rectal atresia with a fistula, as these infants may not require neonatal surgery, leaving 140 infants for analysis. Characteristics of the sample separated by era (Table 1) are reported for the whole sample, the sample excluding large bowel atresia (i.e., including small bowel atresia and gastroschisis), and including only gastroschisis. As a whole, infants in the two eras did not differ by BW, GA, or sex. The mean (SD) birth GA fell into the late preterm/early term range [20,21]. The type of congenital gastrointestinal conditions differed between the eras, with the DM era having more infants with small bowel atresia and fewer with large bowel atresia.

Table 2 delineates the type of enteral feedings received during hospitalization. Feeding categories were non-exclusive, with many infants receiving multiple types of feeds (MOM, DM, or formula) during their hospitalization. As DM was weaned prior to discharge to the planned at-home feeding regimen, no infants received DM in the 48 h prior to discharge. In the first 30 days, for the whole cohort, infants received similar amounts of HM overall, with percentage of MOM in the pre-DM era exactly equal to MOM + DM in the DM era. DM use was relatively infrequent in the DM era (5.7% in the DM group), with a vast majority of infants receiving MOM (88.6% in the DM group) in the first 30 days in both eras. Formula use in the DM era was still common, likely reflecting infants with short length of stay who were supplemented with formula instead of DM (which is not available after discharge) when planning for prompt discharge.

It was noted that fewer infants received formula in the 48 h prior to discharge in the DM era, a drop of nearly 20% for the cohort as a whole (*p* = 0.03), suggesting that the aforementioned use of formula in the first 30 days was only intermittent as a supplement or a bridge to MOM, not to replace MOM.

In-hospital outcomes (Table 3) did not differ between eras for the group as a whole. When large bowel atresias were excluded, hospital LOS and CL days were 10 and 8 days shorter, respectively, in the DM era. Although not reaching statistical significance (*p* = 0.10), infants in the DM era were discharged at 1.2 weeks younger corrected GA (41.1 vs. 42.3 weeks). When the infants at highest risk of feeding intolerance (gastroschisis) were analyzed separately, statistical significance was lost, likely due to low numbers (total n of 44.) Trends remained, however, with LOS 15 days shorter (*p* = 0.10) and central line days 11 days shorter (*p* = 0.07) in the DM era. NEC was rare overall (4 in pre-DM and 2 in DM era) and did not differ between eras in any analysis.

## 4. Discussion

This retrospective study suggests that there is a potential benefit in offering post-operative DM to reduce the LOS and central line days in a subset of the late preterm/term congenital GI malformation population with gastroschisis and small bowel atresias Although not reaching statistical significance, infants in the DM era were discharged at 1.2 weeks younger corrected GA, a clinically significant value not likely explained by the birth GA that was only a few days older in the DM era (*p* = 0.07, 37 vs. 36.3 weeks). No obvious benefit was seen when infants with colonic/rectal atresias were included. The results are scientifically plausible—dysmotility and feeding intolerance frequently feature in these “high-risk” diagnoses (small bowel atresias and gastroschisis), in which the bowel may experience prolonged periods of distension and/or amniotic fluid exposure prenatally [3]. In contrast, infants with large bowel atresias typically present with distension post-natally and often have rapid return of bowel function after receiving a diverting colostomy [15], reflected in the cohort’s longer LOS when infants with large bowel atresias were removed from the analyses (Table 3).

It is unclear for the small bowel atresia and gastroschisis infants if the DM itself is beneficial, as the number receiving DM was small, or if it was the lower rates of formula feeding with accompanying higher rates of MOM feeding around the time of discharge that might have facilitated a decreased LOS, or a combination of the two. Although studies are limited, previous authors have found that offering DM can be associated with higher rates of MOM provision [22,23]. In a retrospective study of 163 infants, Shinnick et al. reported that an exclusively HM diet in a neonatal surgical population consisting mostly of infants with gastroschisis and intestinal atresia was associated with decreased LOS, PN days, and days to full enteral feeds; a mixed diet consisting of partial HM/partial formula did not show similar benefits [7]. The authors report that “very few” of the infants received DM in this cohort, whose mean GA fell into the late preterm range and included infants >1250 g, but no further information was reported. Gulack et al. reported in a retrospective study that the use of HM, defined as MOM or DM, vs. formula resulted in shorter hospital LOS in infants with gastroschisis, but data was not available to differentiate between MOM vs. DM. Given the study time frame of 1997–2012, when DM use was less common even in preterm populations, it is likely that the majority of this HM was MOM, not DM [24]. Kohler et al. reported shorter time to full feeds with exclusive HM vs. mixed diet or exclusive formula in a retrospective gastroschisis study in a similar time frame to Gulack; DM use was not reported [6].

In keeping with our findings of lower rates of formula feeding at NICU discharge in the DM era, previous studies have linked HM, but not DM specifically, to improved outcomes in the gastroschisis and small bowel atresia population [5,6,7,8]. Growth factors such as epidermal growth factor in HM may promote small bowel epithelial growth [25] and gut barrier protection [16]; HM fed infants demonstrate decreased intestinal permeability, which can protect against pathogens in a fragile gut [26]. The milk microbiome can also modulate epithelial cells and promotes a healthy gut microbial environment [16], and bioactive components such as lactoferrin, immunoglobulins and oligosaccharides have anti-inflammatory and prebiotic properties that could promote improved feeding tolerance. Although these components and others are postulated to mediate the myriad of benefits of HM and specifically MOM in the neonatal population, DM has an altered composition. Many, but not all, growth factors and bioactive components are reduced while all living cells are eradicated with pasteurization and processing [27], which likely explains why DM has not been shown to improve morbidities like sepsis in the same way that MOM does [25]. The benefits associated with the DM era in our study could simply be due to less exposure to formula, which can increase intestinal permeability and inflammation. This formula avoidance is postulated to partially explain the reduction in NEC in preterm infants with DM use [16,28], and could also explain the findings of Kohler et al. and Shinnick et al., who reported that exclusive HM diets had better outcomes than even infants fed a majority HM, but some formula, in the congenital GI malformation population [5,6,7]. However, the dose of MOM vs. DM may be important given the aforementioned differences, and researchers should not combine MOM and DM together as “HM” [16]. It is important to study differences in DM outcomes according to the dose of MOM fed.

The strengths of our study were the utilization of a high volume single center with consistent feeding protocols between eras in a relatively short time frame. Access to records that differentiated between MOM and DM was a particular strength of our study that addresses a common limitation of previous research in this area. This is the first study we are aware of specifically assessing DM to supplement MOM in the congenital GI malformation surgical population. Limitations include a retrospective study with a relatively small n, although in keeping with other cohorts given diagnostic rarity [6,7]. The low NEC rates in both eras limited our ability to detect any differences with DM. Although there were no other significant practice changes implemented in our NICU between the two eras, it is possible that other confounding factors may have been responsible for the differences seen between the eras. Finally, our data included the type of feed, but not the proportion of each type of feed, so we were unable to determine doses of MOM or DM versus formula or exclusivity of MOM.

Our study provides the first evidence of potential benefit of offering post-operative DM when MOM is limited in supply in the congenital GI malformation population, specifically for infants with gastroschisis and small bowel atresia. More studies of DM in this population are warranted, and could include additional congenital GI conditions with motility challenges such as omphalocele and Hirschprung’s disease. Although DM is a costly product, it has been shown to be cost effective in the very preterm population based on reductions in NEC [29]. In addition, if offering DM is associated with increased rates of MOM feeding, then this practice could have significant short and long-term secondary benefits. A randomized controlled trial of DM vs. formula to supplement inadequate MOM in this population would provide further evidence and may help address important questions such as how long to continue DM post-operatively, when to introduce formula if needed, and the cost-effectiveness of DM. Finally, centers with low baseline rates of MOM feedings may see larger outcome differences with the use of DM compared to our cohort, in which MOM use is relatively prevalent.

## 5. Conclusions

In conclusion, a practice change to offer DM was associated with shorter LOS and central line days in a late preterm and term population with gastroschisis and small bowel atresia, providing the first evidence for the potential use of a limited resource in this high-risk surgical population when MOM volumes are insufficient. More studies are needed to confirm our findings at other centers as well as to delineate the duration of DM therapy and its cost effectiveness.

## Figures and Tables

**Table 1:** Characteristics of the sample.

Median (IQR) or *n* (%)	Pre-DM Era	DM Era	*p* Value, Mann Whitney *u* Test or Chi-Square
**Whole Sample**	***n*** **= 70**	***n*** **= 70**	
Birthweight (grams)	2790 (2388, 3310)	2810 (2423, 3161)	0.83
Birth length (cm)	47.5 (44.0, 50.0) *n* = 45	47.0 (45.0, 50.0) *n* = 65	0.87
Birth HC (cm)	32.8 (31.4, 33.5)	33.0 (32.0, 34.0) *n* = 69	0.43
Birth GA (weeks)	37 (36, 38)	37 (36, 38)	0.80
Male sex	44 (62.9%)	40 (57.1%)	0.49
Multiple GI malformations (i.e., gastroschisis + atresia)	5 (7%)	7 (10%)	0.36
Gastroschisis	25 (35.7%)	19 (27.1%)	0.01 *
Small bowel atresia (duodenal, jejunal, ileal)	22 (31.4%)	39 (55.7%)	
Rectal/colonic atresia without fistula	23 (32.9%)	12 (17.1%)	
**Excluding Large Bowel (Rectal/Colonic) Atresia**	***n*** **= 47**	***n*** **= 58**	
Birthweight (grams)	2690 (2300, 3070)	2715 (2423, 3159)	0.45
Birth length (cm)	46.8 (44.0, 50.0) *n* = 30	47.0 (45.0, 50.0) *n* = 53	0.36
Birth HC (cm)	32.5 (31.0, 33.0)	33.0 (32.0, 34.0) *n* = 57	0.13
Birth GA (weeks)	36.3 (35.0, 38.0)	37.0 (36.0, 38.0)	0.07
Male sex	27 (57.4%)	32 (55.2%)	0.82
Gastroschisis	25 (53.2%)	19 (32.8%)	0.04 *
Small bowel atresia	22 (46.8%)	39 (67.2%)	
**Only Gastroschisis**	***n* = 25**	***n* = 19**	
Birthweight (grams)	2480 (2205, 2918)	2640 (2430, 3080)	0.15
Birth length (cm)	45.0 (42.9, 48.8) *n* = 16	45.0 (43.0, 49.0)	0.94
Birth HC (cm)	32.0 (31.0, 33.0)	33.0 (32.0, 34.0)	0.03 *
Birth GA (weeks)	36.0 (35.0, 37.0)	37.0 (36.0, 37.0)	0.02 *
Male sex	15 (60.0%)	10 (52.6%)	0.63

IQR: intraquartile range; DM: donor milk; HC: head circumference; GA: gestational age in completed weeks; GI: gastrointestinal. * *p* < 0.05.

**Table 2:** Enteral feeding during hospitalization.

*n* (%)		Pre-DM Era	DM Era	*p* Value, Chi-Square
**Whole Sample**		***n*** **= 70**	***n*** **= 70**	
**Received during the first 30 days of life**				
	MOM	66 (94.3%)	62 (88.6%)	0.23
	DM	0	4 (5.7%)	0.04 *
	Formula	37 (52.9%)	34 (48.6%)	0.61
**Received in the 48 h prior to discharge**				
	MOM	50 (71.4%)	56 (80.0%)	0.24
	Formula	35 (50.0%)	22 (31.4%)	0.03 *
**Excluding Large Bowel (Rectal/Colonic) Atresia**		***n*** **= 47**	***n*** **= 58**	
**Received during the first 30 days of life**				
	MOM	45 (95.7%)	51 (87.9%)	0.16
	DM	0	4 (6.9%)	0.07
	Formula	22 (46.8%)	28 (48.2%)	0.88
**Received in the 48 h prior to discharge**				
	MOM	32 (68.1%)	47 (81.0%)	0.13
	Formula	24 (51.1%)	19 (32.8%)	0.06
**Only Gastroschisis**		***n* = 25**	***n* = 19**	
**Received during the first 30 days of life**				
	MOM	25 (100%)	15 (78.9%)	0.02 *
	DM	0	1 (5.3%)	0.25
	Formula	14 (56.0%)	13 (68.4%)	0.40
**Received in the 48 h prior to discharge**				
	MOM	15 (60.0%)	14 (73.7%)	0.34
	Formula	15 (60.0%)	10 (52.6%)	0.63

DM: donor milk; MOM: mother’s own milk. * *p* < 0.05.

**Table 3:** Outcomes.

Median (IQR) or *n* (%)	Pre-DM Era	DM Era	*p* Value, Mann Whitney—*u* Test or Chi-Square
**Whole Sample**	***n*** **= 70**	***n*** **= 70**	
Hospital LOS (days)	28.0 (17.8, 54.3)	25.0 (16.75, 45.0)	0.26
PN days	11.0 (6.0, 23.0)	14.0 (8.0, 24.3)	0.38
Central line days	21.0 (6.0, 44.5)	18.0 (10.8, 29.3)	0.34
NEC	4 (1 medical, 3 surgical) (5.7%)	2 (surgical) (2.9%)	0.40
Culture positive bloodstream infection	6 (11.4%)	8 (8.6%)	0.57
Death prior to discharge	1 (1.4%)	1 (1.4%)	1.00
Discharge weight (grams)	3169 (2734, 3532)	3056 (2625, 3560) *n* = 69	0.31
Discharge HC (cm)	33.5 (32.6, 34.9) *n* = 52	33.3 (32.5, 34.7) *n* = 36	0.57
Discharge CGA (weeks)	41.1 (40.1, 44.7)	41.1 (39.6, 44.2)	0.29
**Excluding Large Bowel (Rectal/Colonic) Atresia**	***n*** **= 47**	***n*** **= 58**	
Hospital LOS (days)	35.0 (22.0, 76.0)	25.0 (18.0, 43.5)	0.01 *
PN days	14.5 (8.8, 26.8)	14.5 (9.0, 24.3)	0.82
Central line days	28.0 (15.0, 71.0)	20.0 (12.5, 29.3)	0.01 *
NEC	4 (1 medical, 3 surgical) (8.5%)	2 (both surgical) (3.4%)	0.27
Culture positive bloodstream infection	8 (17%)	4 (6.9%)	0.11
Death prior to discharge	0	1 (1.7%)	0.37
Discharge weight (grams)	3135 (2705, 3670)	3125 (2668, 3680)	0.54
Discharge HC (cm)	33.8 (32.2, 34.6) *n* = 34	34.0 (32.5, 35.0) *n* = 27	0.77
Discharge GA (weeks)	42.3 (40.1, 47.9)	41.1 (39.6, 44.6)	0.10
**Only gastroschisis**	***n*** **= 25**	***n*** **= 19**	
Hospital LOS (days)	44.0 (34.5, 77.0)	29.0 (26.0, 52.0)	0.10
PN days	15.0 (10.0, 29.0)	21.0 (12.0, 34.0)	0.64
Central line days	36.0 (26.0, 73.5)	25.0 (21.0, 45.0)	0.07
NEC	3 (1 medical, 2 surgical) (12.0%)	1 surgical (5.3%)	0.44
Culture positive bloodstream infection	4 (16%)	1 (5.3%)	0.27
Death prior to discharge	0	0	
Discharge weight (grams)	3100 (2698, 3625)	2810 (2625, 3865)	0.74
Discharge HC (cm)	33.0 (32.0, 34.5) *n* = 18	33.3 (32.5, 35.0) *n* = 8	0.34
Discharge CGA (weeks)	43.0 (40.3, 50.0)	41.3 (40.6, 44.9)	0.33

IQR: intraquartile range; DM: donor milk; LOS: length of stay; PN: parenteral nutrition; NEC: necrotizing enterocolitis; HC: head circumference; CGA: corrected gestational age. * *p* < 0.05.

## Abbreviations

BW	birth weight
DM	donor human milk
GA	gestational age
LOS	length of stay
MOM	mother’s own milk
NEC	necrotizing enterocolitis
PN	parenteral nutrition
